# Highly Selective Detection of Metronidazole by Self-Assembly via 0D/2D N–C QDs/g-C_3_N_4_ Nanocomposites Through FRET Mechanism

**DOI:** 10.1186/s11671-020-3294-2

**Published:** 2020-04-19

**Authors:** Shan Wang, Jing Fu, Fang Zhang, Ruirui Huan, Ting Liu, Xingguo Zeng

**Affiliations:** School of Chemistry and Chemical Engineering, Xianyang Normal University, Xianyang, 712000 People’s Republic of China

**Keywords:** FRET, N–C QDs, g-C_3_N_4_ nanosheets, Metronidazole, Sensor

## Abstract

**Abstract:**

A 0D/2D (0-dimensional/2-dimensional) nanostructure was designed by self-assembly of N–C QDs and carboxylated g-C_3_N_4_ nanosheets and used as a fluorescence resonance energy transfer (FRET) fluorescent sensor. The N–C QDs/g-C_3_N_4_ nanosheets were synthesized via the amino group on the N–C QD surface and the –COOH of the carboxylated g-C_3_N_4_ nanosheets. The mechanism of detection of metronidazole (MNZ) by N–C QDs/g-C_3_N_4_ nanocomposites is based on FRET between negatively charged N–QDs and positively charged carboxylated g-C_3_N_4_ nanoparticles. N–C QDs/g-C_3_N_4_ nanostructures displayed good responses for the detection of MNZ at normal temperature and pressure. The decrease in the fluorescence intensity showed a good linear relationship to MNZ concentration within 0–2.6 × 10^−5^ mol/L, and the detection limit was 0.66 μM. The novel FRET sensor will have a great potential in clinical analysis and biological studies.

**Graphical Abstract:**

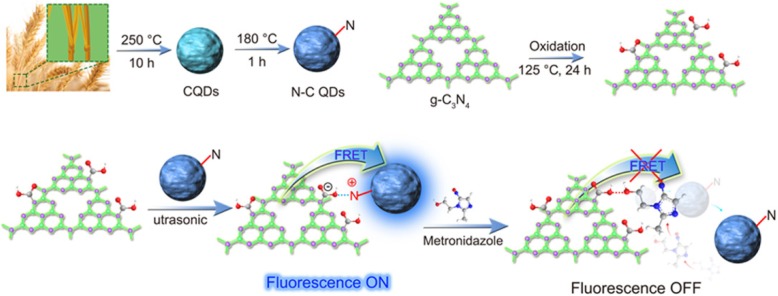

## Background

Two-dimensional nanomaterials (such as graphene, WS_2_, g-C_3_N_4_, MoS_2_, and MnO_2_) have been widely used in the field of sensing because of its unique characteristics such as atomic layer thickness and special physical, chemical, and biological compatibility [1]. In recent years, a two-dimensional nanostructure-based photo-chemical (PEC) detection strategy has been widely concerned, since the excitation light source and the electrical readout signal in the method are part of a different energy form and have a low background signal and a high sensitivity. g-C_3_N_4_ is environment-friendly and exhibit a lower sensitivity in the photo-sensing system [2–6]. Nanostructures (as a multiphase solid nanomaterials) have been significantly expanded to include systems composed of obviously different components (such as quantum dots and nanosheets 0D/2D) [7–9]. Compared with heavy metal semiconductors, carbon quantum dots (C quantum dots) have unique advantages (such as non-toxicity, easy modification, good stability, and ecological friendliness with other materials) [10–12]. More importantly, the structural engineering of 2D-g-C_3_N_4_ nanosheets to zero-dimensional quantum dots will make g-C_3_N_4_ have more attractive electronic structure, optical absorption capacity, and favorable band gap caused by unique quantum limiting effect [13–15]. Combination of C QDs with g-C_3_N_4_ nanosheets for the preparation of 0D/2D nanostructure is conducive for FRET in the metronidazole measurement that was designed.

In this paper, a simple and green self-assembly strategy for electrostatic self-assembly is developed. The heterostructures of nanosheets g-C_3_N_4_ and N–C QDs with close heterogeneous interfaces are constructed, in which g-C_3_N_4_ quantum dots with negative charges and N–QDs with positive charges are used as the building blocks. Given that the g-C_3_N_4_ and the N–C QDs realize the reasonable energy band through intelligent structure modulation, the unique 0D/2D integration mode is realized, the g-C_3_N_4_ quantum dot with the rich active center is reasonably combined with the N–C QDs, and the optical performance of the g-C_3_N_4_ nanosheets/N–C QD composite material is obviously promoted. A FRET strategy between N–C QDs and carboxylic g-C_3_N_4_ nanosheets is designed to promote electron/hole separation. The photocurrent of the nanostructure is greatly increased [16]. The positively charged N–QDs form FRET assemblies with negatively charged carboxylic g-C_3_N_4_ nanosheets, leading to strong quenching of fluorescence emission.

## Methods

### Materials

N–QDs were prepared by hydrothermal method [17, 18], and g-C_3_N_4_ nanosheets were prepared by the method of the laboratory [19]. Metronidazole was received from Shanghai Shenbo Chemical Co., Ltd. (Shanghai, China). Other reagents were of analytical reagent grade and bought from Aladdin reagent co., Ltd. Double distilled water was used in all the experiments.

### Instruments

HRTEM were gotten from a JEM-2100F microscope (Hitachi, Japan). SEM was acquired from an S-4800 microscope (Hitachi, Japan).Absorption was carried out on a 650 UV-vis photometer (PerkinElmer, USA).Fluorescence measurements were carried out on an F-4600 spectrophotometer (Hitachi, Japan).

### Preparation of carboxylated g-C_3_N_4_ Nanosheets

The g-C_3_N_4_ nanostructure was prepared by heating urea to 550 °C for 3 h. The resulting yellow aggregates were carefully ground into powder with hammer and mortar. The large g-C_3_N_4_ nanostructure was dispersed into ultrapure water for 12 h, and the suspension was centrifuged for 10 min (4 500 r/min) to obtain the supernatant of g-C_3_N_4_ nanosheet. g-C_3_N_4_ nanosheets were obtained by centrifugation at 10,000 r/min for 10 min. The precipitate was finally dried in a vacuum oven at 50 °C [20].

g-C_3_N_4_ nanosheets (2 g) were placed into the beaker and added with a small amount of distilled water. The solution was subjected to ultrasonication for several hours to produce a uniform emulsion. The bottom impurities were removed, and the liquid was dried to obtain solid g-C_3_N_4_ nanosheets. g-C_3_N_4_ nanosheets (1 g) were weighed, placed into 150 mL round bottom flask, and added with 100 mL of 5 mol/L HNO_3_ solution. The reflux device was installed in sequence and placed in a thermostatic oil bath filled with paraffin oil. The temperature was set to 125 °C, and reflux was kept at this temperature for one day. The liquid was washed with low-speed table centrifuge to neutral pH value after being cooled to room temperature. The precipitate was dried in an air dryer at 35 °C to obtain carboxylated g-C_3_N_4_ nanosheets.

### Preparation of N–C QDs

Wheat straw was crushed, dried for 6 h at 80 °C, and added with 3 mol/L NaOH solution in a 100 mL bottle. NaOH solution (35 mL) was added to 0.5 g of dried straw powder and continuously stirred for 10 min. The stirred solution was poured into 50 mL polytetrafluoroethylene lining, which was placed into a high temperature reactor. The temperature was adjusted to 250 °C in a blast drying box for 10 h. The samples was removed, cooled to room temperature, and filtered for 30 min.

About 0.175 mL of 2,6,6-tetramethylpiperidine solution was added to 30 mL of carbon quantum dot solution prepared in the previous step. The solution was mixed evenly by stirring under magnetic force for 10 min. The mixed solution was transferred to 50 mL polytetrafluoroethylene lining, and the liner was placed in a high-temperature reactor. The modified carbon quantum dot solution was obtained by heating up to 180 °C in the oven for an hour and then cooled naturally to room temperature.

### Preparation of N–C QDs/g-C_3_N_4_ Nanostructures

N–C QDs-functionalized g-C_3_N_4_ nanostructures were prepared. Equal volumes (32 mL) of ethanol and ultrapure water were mixed. The aqueous ethanol solution was added with 0.12 g of g-C_3_N_4_ nanosheets and 2.4 mg of the aminated C–QDs under vigorous stirring. The sample was washed twice with ultrapure water, twice under centrifugation, and dried at 50 °C in a vacuum oven.

### Fluorescence Sensing of Metronidazole (MNZ)

The corresponding suspension was transferred into a 100 mL Teflon-lined autoclave after sonication for 30 min and then heated at 180 °C for 4 h for fluorescence sensing of MNZ. The N–C QDs/g-C_3_N_4_ nanostructures were washed. Fluorescence spectra were recorded after fixed amounts of g-C_3_N_4_ nanosheets (500 μL) were added into the sample containing different MNZ concentrations. The MNZ level in samples was determined according to the following steps. Different concentrations of MNZ were mixed with 500 μL of the g-C_3_N_4_ nanosheet from the stock dispersion and 2.0 mL of the phosphate buffer solution (0.04 mol/L). The solution was transferred for luminescence measurements with an excitation wavelength of 330 nm after standing for 5 min. The excitation wavelength (*λ*_ex_) was 330 nm for all measurements of g-C_3_N_4_ when its corresponding emission wavelength (*λ*_em_) was monitored from 360–600 nm. The excitation and emission slit widths for the g-C_3_N_4_ nanosheet were set at 5 nm for all measurements.

## Results and Discussion

### Fourier-Transform Infrared Spectroscopy of C QDs, N–C QDs, and g-C_3_N_4_:N–C QD composites

**Fig. 1:**
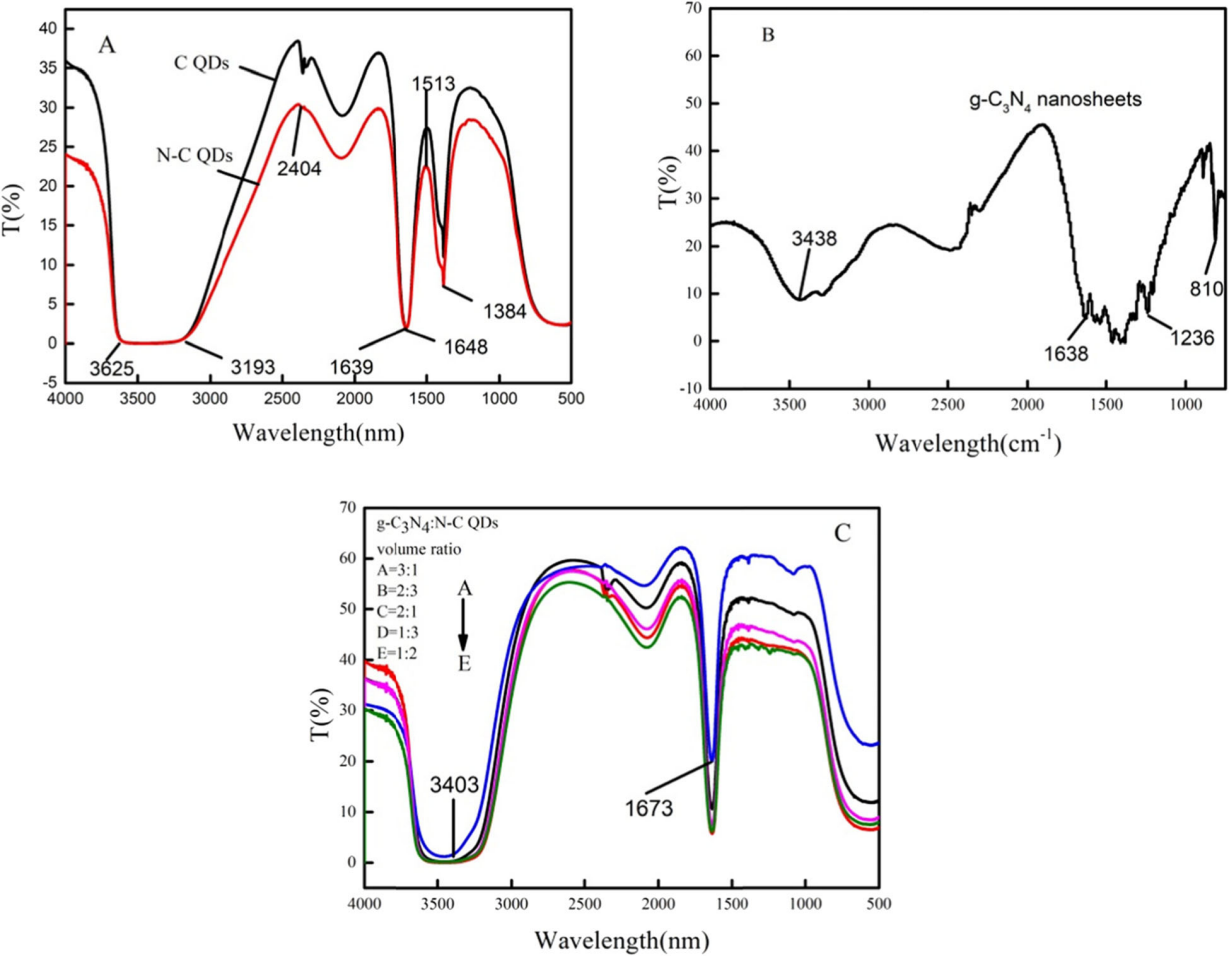
FTIR of C QDs and N-C QDs (**a**), g-C_3_N_4_ nanosheets (**b**) and g-C_3_N_4_: N-C QDs composites (**c**)

The peak of the g-C_3_N_4_ nanosheet is due to the stretching vibration of the defect center terminal-NH_x_ group bond and the stretching vibration of the physical adsorption water (O–H) between 3400 and 3000 cm^−1^(Fig. 1a). The characteristic bands at 1236 and 1638 cm^−1^ belong to a typical C–N and C=N heterocyclic stretch vibration mode, respectively, whereas the characteristic band at 810 cm^−1^ is attributed to the triazine unit breathing pattern. As shown in the figure, the bands at 810, 1638, and 3438 cm^−1^ are the three characteristic absorption peaks of triazine heterocycle, C–N bond, and N–H bond of g-C_3_N_4_. The telescopic vibration of C–N bond corresponds to the range of 1236–1635 cm^−1^.

As shown in the figure, in the infrared spectrum of C QDs, the band at 1639 cm^−1^ corresponds to the characteristic absorption peak of –C=O and is assigned to the telescopic vibration of O–H and N–H within 3193–3625 cm^−1^. Moreover, the bands at 2500–2000 cm^−1^ belong to the –NCO group. Hence, the existence of oxygen-containing groups, –OH and –NH_2,_ confirms the water solubility of C QD solution. The fluorescence emission performance of C QDs can be improved because –OH is an electron donor group. The infrared spectrum of the C QDs/g-C_3_N_4_ nanosheet (Fig. 1b) was similar to the infrared spectrum of the bare C QDs because the peak position was substantially the same. The characteristic absorption peaks at 3182, 2404, 1648, 1513, 1517, 1390, and 1183 cm^−1^ in the infrared spectra of N–C QDs are attributed to C–OH, C–H, C=O, N–H, C–N, and C–O–C, respectively, indicating that the synthesized N–C QD surface has many hydrophilic groups and a strong hydrophilicity. The direction of the infrared was the same when the C QDs and the g-C_3_N_4_ nanosheets of different proportions were combined (Fig. 1c). The peaks at 3404 cm^−1^ suggest the existence of –NH_2_ and –NH_3_^+^ at the aminated C QDs, whereas the peaks at 1673 cm^−1^ correspond to the stretching vibrations of amide I, C=O, amide II, N–H, and amide III, C–N groups. The transmittance detected by infrared spectrometer was the best when the volume ratio of g-C_3_N_4_:N–C QD composites was 3:1.

### Optical Characteristics of C QDs, N–C QDs, and g-C_3_N_4_:N–C QD composites

**Fig. 2:**
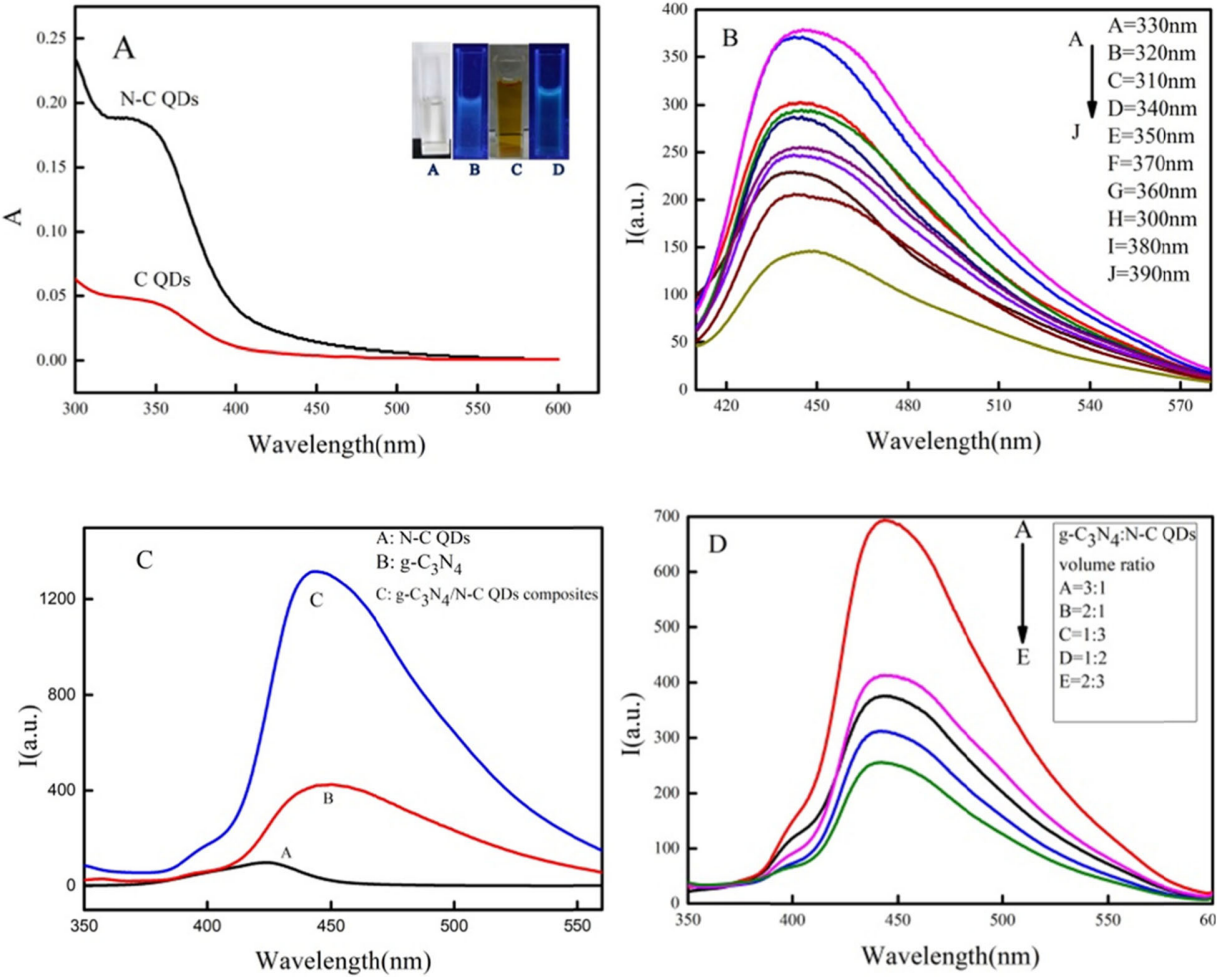
Optical characteristics of C QDs, N-C QDs, g-C_3_N_4_: N–C QDs composites (**a** Uv-vis properties of C QDs, N-C QDs. **b** The excite properties of composites. **c** The properties of QDs. **d** The optimal proportion of composites)

**Fig. 3:**
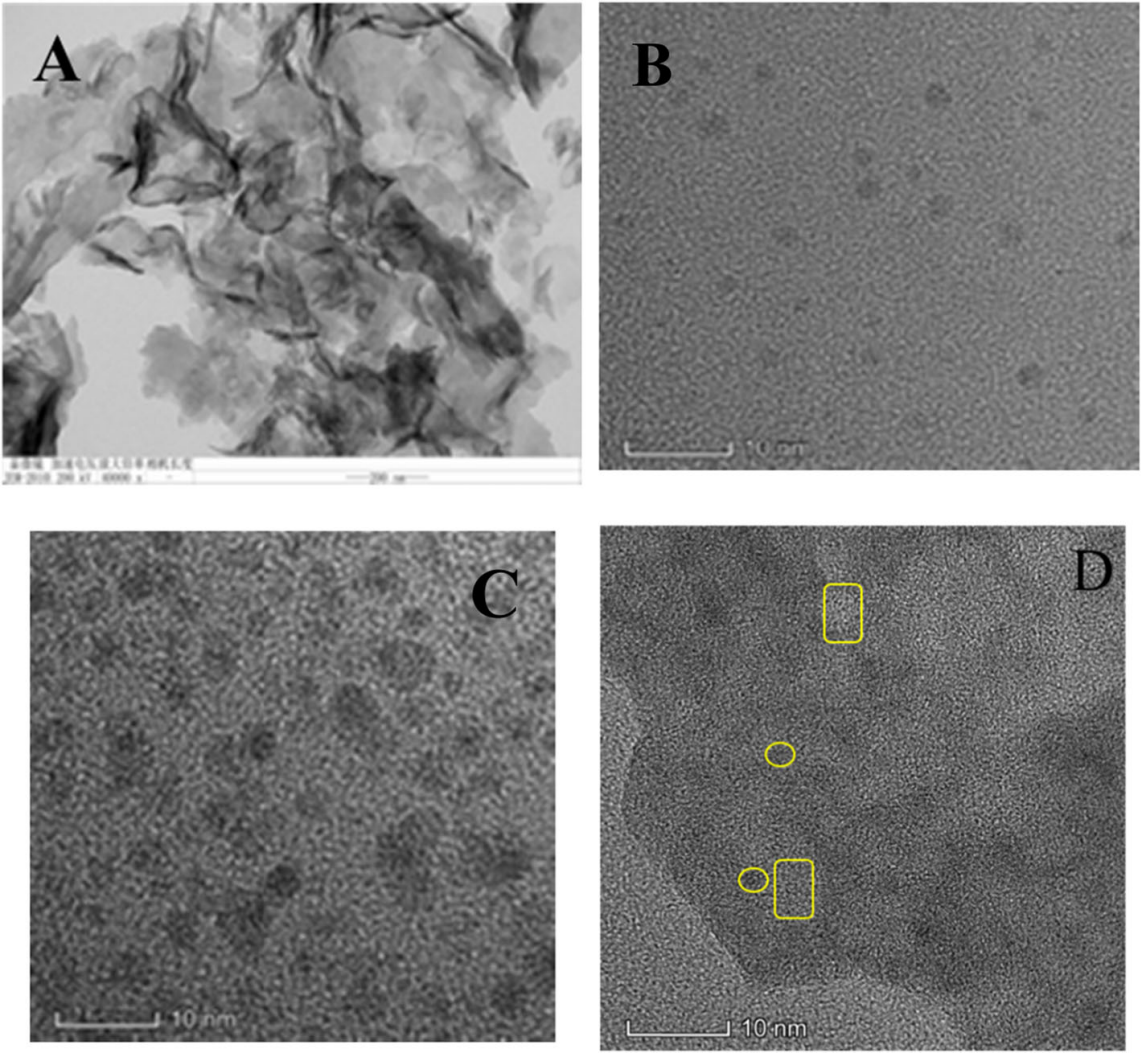
HRTEM images of **a** g-C_3_N_4_ nanosheets, **b** C QDs, **c** N–C QDs and **d** N–C QDs/g-C_3_N_4_ nanocomposites

The properties of the prepared QDs and nanocomposites were characterized using various types of instruments. Optical characterization of C QDs was performed with ultraviolet (UV)–visible (Vis) absorption and fluorescence spectroscopies, and their spectra are shown in Fig. 2a. The absorption peaks were located at about 344 nm in the UV–Vis absorption spectra of C QDs that was close to the maximum excitation peak (red line) of 330 nm in the fluorescence spectrum of g-C_3_N_4_ nanosheets:N–C QD composites (Fig. 2b), implying the high recombination possibility of electron–hole pairs. The UV–Vis absorption spectrum of N–C QDs is higher at 344 nm. As shown in Fig. 3a, the C QDs were transparent (a) under natural light but became blue when irradiated with 365 nm UV light (b), thereby confirming the blue fluorescence properties of C QDs. The color of the solution was changed from transparent to light yellow under natural light (c) when C QDs was modified by nitrogen hybridization. The N–C QD solution was bluer than that of C QDs (d) when irradiated with 365 nm UV light. The emission spectrum of g-C_3_N_4_:N–C QD composites is shown in Fig. 2b when the composite was excited at the excitation wavelengths of 310, 320, 330, 340, 350, 360, 370, 380, and 390 nm. Result was determined that 330 nm was the optimal excitation wavelength of g-C_3_N_4_:N–C QDs composites. In addition, Fig. 2b shows that the position of the emission wavelength of the g-C_3_N_4_:N–C QD composites does not substantially change as the excitation wavelength changes but only with the increase or decrease in the fluorescence intensity. In addition, Fig. 3c shows that the fluorescence intensities of the N–C QDs (A), g-C_3_N_4_ nanosheets (B), and g-C_3_N_4_:N–C QD composites (C) were increased in sequence when excited by the same excitation wavelength, and the fluorescence resonance energy transfer occurs between the N–C QDs and g-C_3_N_4_ nanosheets. The intensity of its fluorescence spectrum at the excitation wavelength of 330 nm was the strongest (Fig. 2d) when the volume ratio of g-C_3_N_4_ nanosheets:N–C QDs was 3:1.

### Transmission Electron Microscopy

Figure 3 shows the high-resolution transmission electron microscopy images of g-C_3_N_4_ nanosheets, C QDs, N–C QDs, and the g-C_3_N_4_:N–C QD composite nanostructures. Figure 3a shows that the as-synthesized g-C_3_N_4_ nanosheets displayed planar sheet-like morphology with an irregular shape. Moreover, the synthesized C QDs exhibited regular well-dispersed spherical nanoparticles with a size distribution range from 3 nm to 5 nm (Fig. 3b). Nitrogen-modified C QDs showed spherical shape but were slightly different in size (Fig. 3c). The lattice in the pristine N–C QDs (Fig. 3c) could be obviously achieved after the formation of N–CQDs/g-C_3_N_4_ nanocomposites (Fig. 3d). The carboxylic g-C_3_N_4_ nanosheet aqueous solution was notably negatively charged because of the carboxyl modification. In this regard, the negatively charged g-C_3_N_4_ QDs was reasonably speculated to be spontaneous and uniformly self-assembled on the 2D framework of positively charged N–C QDs with intimate interfacial contact under pronounced electrostatic interaction on the basis of the surface charge properties of g-C_3_N_4_ QDs and C QDs as assembly units.

### X-ray Photoelectron Spectroscopy

**Fig. 4:**
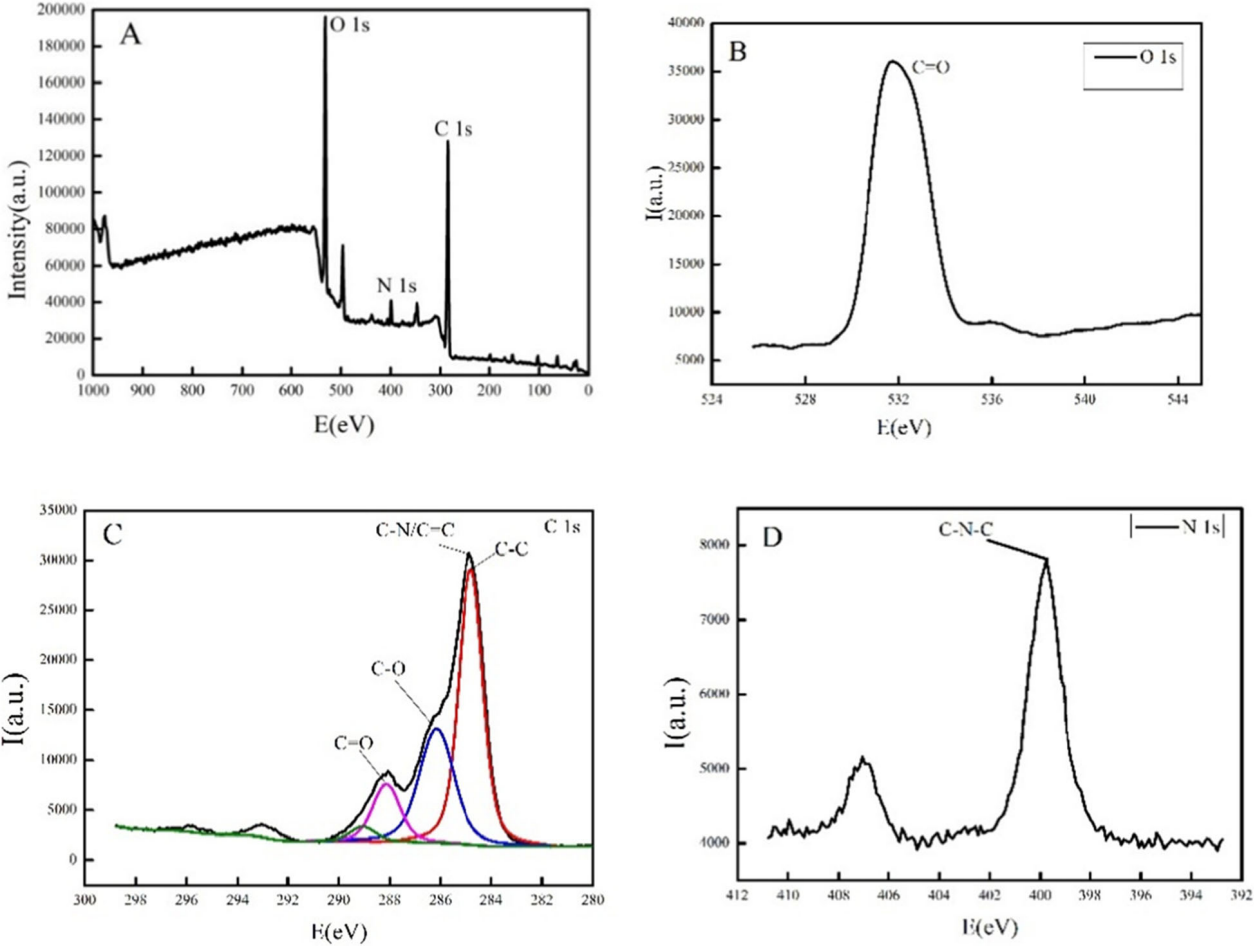
The X-ray photoelectron spectroscopy of g-C_3_N_4_/N–C QDs composites

Figure 4a presents the X-ray photoelectron spectra (XPS) of g-C_3_N_4_/N–C QD composites. g-C_3_N_4_ /N–C QD composites contain three elements, namely, carbon, nitrogen, and oxygen, with corresponding peak positions of 285 (C 1 s), 399.3 (N 1 s), and 531.51 eV (O 1 s). Figure 4b shows the existence of oxygen in g-C_3_N_4_/N–C QD composites and C=O bond (531.9 eV). Figure 4c shows the existence of carbon in g-C_3_N_4_/N–C QD composites, which are C–C bond (284.8 eV), C–N/C=C (284.87 eV), C–O bond (286.16 eV), and C=O bond (288.14 eV). Figure 4d shows the partial peak spectrum of nitrogen, and the energy of 399.8 eV corresponds to the C–N–C bond. These results are consistent with the infrared spectra. Based on the data of XPS and infrared spectroscopy, abundant functional groups, such as –OH and –NH_2_, which make the surface of g-C_3_N_4_/N–C QD composites introduce defect sites, are present in g-C_3_N_4_/N–C QD composites. These surface defects make them play the function of excitation energy band; thus, g-C_3_N_4_/N–C QD composites have unique fluorescence properties.

### The Fluorescence Properties of the g-C_3_N_4_/N–C QD Composite

#### Effect of pH

**Fig. 5:**
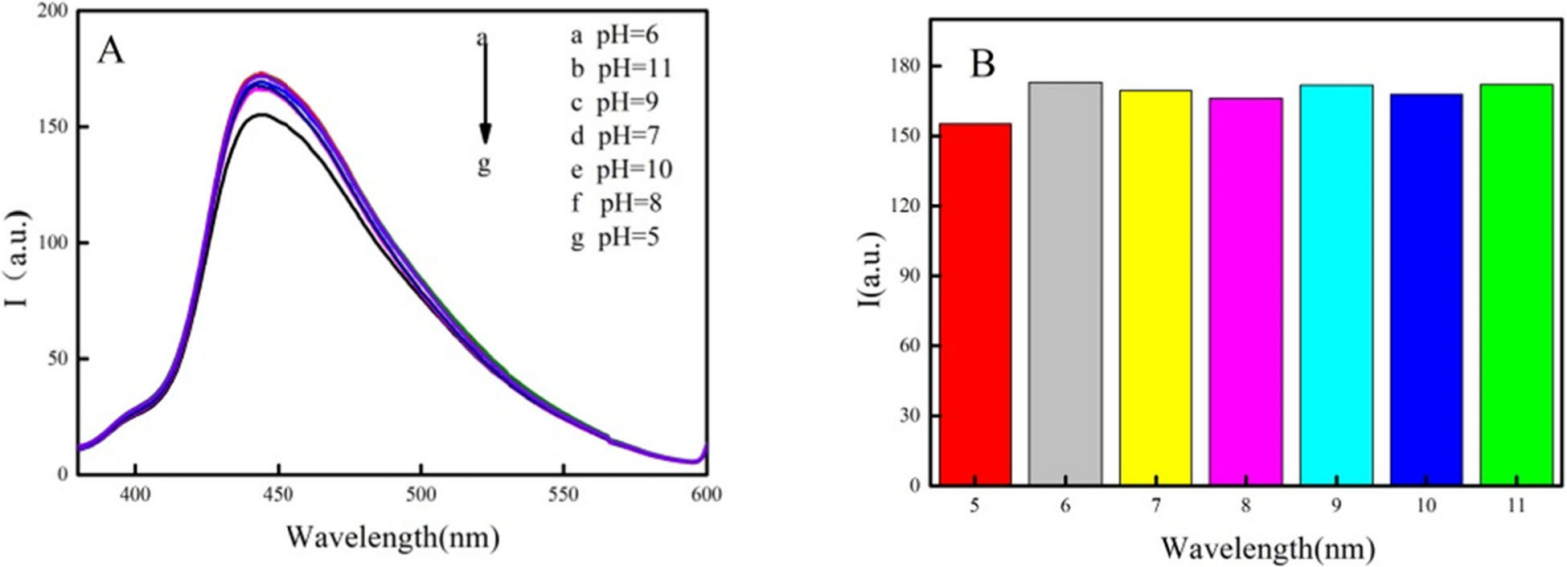
Effect of pH on the detection of MNZ

The relationship between the fluorescence of N–C QDs/g-C_3_N_4_ nanocomposites and pH in the presence of MNZ is shown in Fig. 5a. In this paper, the fluorescence quenching efficiency of MNZ on N–C QDs/g-C_3_N_4_ nanocomposites is defined as *F*_0_/*F*, where *F*_0_ and *F* are the fluorescence intensities of N–C QDs/g-C_3_N_4_ nanocomposites in the presence and absence of MNZ, respectively. When pH increased from 5 to 7, the fluorescence quenching efficiency increased, whereas pH increased further and *F*_0_/*F* decreased. Simultaneously, the effects of different buffer systems on fluorescence quenching efficiency were compared. Figure 5b shows that the quenching efficiency of sodium phosphate buffer system was high. The experimental results show that N–C QDs and carboxyl g-C_3_N_4_ nanosheets combine by self-assembly, thereby interrupting the process of protonation and deprotonation [21]; therefore, the composites are stable in both subacidic and subalkaline systems. This condition may be due to the formation of hydrogen bonds by noncovalent bonds of carboxyl and nitrogen groups on the QDs [22]. Therefore, pH = 7 was selected as the detection condition.

#### Fluorescence Properties of Complex-Quenched MNZ

Several analytical methods have been reported in literature for the detection of MNZ. The most common analysis parameters in a series of literature data, such as the linear range and the LOD, are compared and recovered in Table 1. Most analytical methods for MNZ determination showed limited linear range. These methods were not compared with independent methods in many cases.

**Table 1:** The different fluorescence methods for the detection of melamine

Methods	Probe	Linear range (mol/L)	Detection (mol/L)	References
FRET sensor Electrochemistry Fluorescence Colorimetry Ratiometric fluorescence Fluorescence FRET sensor	C QDs/AuNPs CNT-IL/MIP/GCE C QDs-Hg^2+^ AgNPs AuNCs N–C QDs-Fe^3+^ N–C QDs/g-C_3_N_4_	0.05–0.5 0.4–120 1–20 0.79–7.9 100–8.0 × 10^3^ 2–290 0–2.6 × 10^−5^	0.036 0.11 0.3 0.79 28 0.66 –	[23] [24] [25] [26] [27] [28] This work

In this work, FRET is based on the electrostatic interaction between the N–C QDs and the g-C_3_N_4_ nanosheets. The accuracy, detection limit, linear range, and specificity of this method were studied to validate it. The fluorescence intensity of the g-C_3_N_4_/N–C QD composite at 450 nm was unstable in the first few minutes at the initiation of the addition of MNZ. After about 6 min, the fluorescence intensity of the g-C_3_N_4_/N–C QD composite slowly decreased and then to a minimum, and thereafter almost unchanged. Thus, all fluorescence measurements were carried out within 6 min after adding MNZ.

**Fig. 6:**
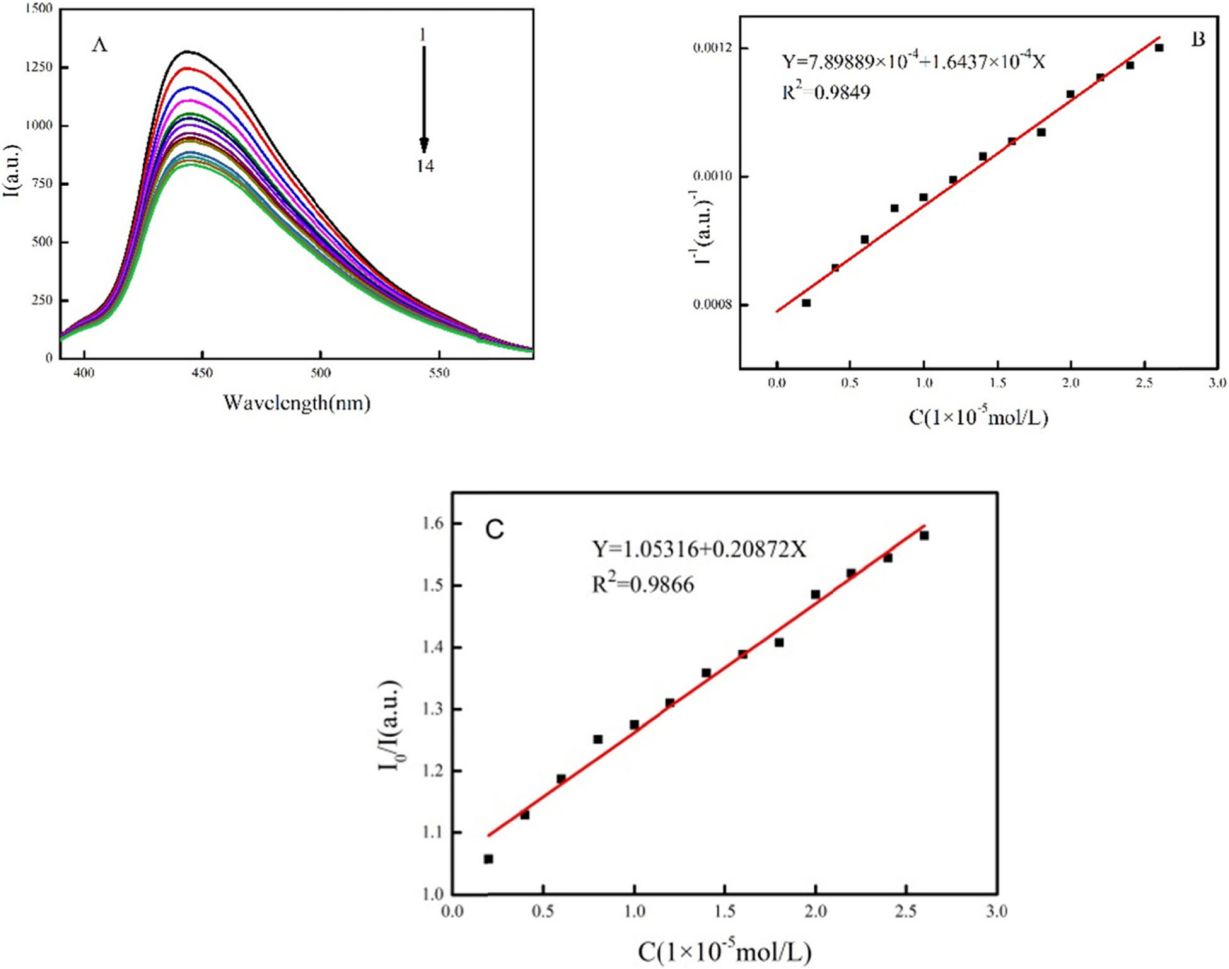
The fluorescence properties of complex quenched MNZ (C_metronidazole_: 0; 0.2 × 10^−5^ mol/L; 0.4 × 10^−5^ mol/L; 0.6 × 10^−5^ mol/L; 0.8 × 10^−5^ mol/L; 1 × 10^−5^ mol/L; 1.2 × 10^−5^ mol/L; 1.4 × 10^−5^ mol/L; 1.6 × 10^−5^ mol/L; 1.8 × 10^−5^ mol/L; 2 × 10^−5^ mol/L; 2.2 × 10^−5^ mol/L; 2.4 × 10^−5^ mol/L; 2.6 × 10^−5^ mol/L)

Figure 6a shows that the fluorescence intensity (450 nm) of the FRET probe was reduced from 1299 (a.u.) to 824 (a.u.) when MNZ was added to the g-C_3_N_4_/N–C QD composite biosensor. Figure 6b shows the fluorescence signal–quenching intensity consistent with different MNZ concentrations, where the fluorescence signal quenching corresponding to those concentrations are 1299, 1230, 1148, 1095, 1042, 1021, 992.4, 958.1, 937.3, 923.4, 874.8, 857.9, 842.1, and 824.2 (a.u.). The intensity of fluorescence quenching is a linear function of the concentration of MNZ in the range from 0 mol/L to 2.6 × 10^−5^ mol/L. The linear equation is *F* = 1.0532 + 0.2087 C (*C* is the MNZ concentration and *F* is the fluorescence intensity) with the correlation coefficient *R*^2^ of 0.9849 (Fig. 6b) .The fluorescence quenching efficiency of the g-C_3_N_4_/N–C QD composite gradually decreased as MNZ concentration increased. The detection limit of MNZ was 0.66 μM (*S*/*N* = 3). The complex fluorescence of the g-C_3_N_4_/N–C QDs was proved to be effectively quenched by the MNZ.

Quenching efficiency is expressed by *Q* = 1 − *F*_q_/*F*_0_, where *F*_q_ is the fluorescence intensity of N–C QDs/g-C_3_N_4_ nanocomposites after quenching, and *F*_0_ is the original fluorescence intensity of N–C QDs/g-C_3_N_4_ nanocomposites. The quenching efficiency changed slightly when the concentration of MNZ increased from 0 mol/L to 2.6 × 10^−5^ mol/L (*Y* = 1.0532 + 0.2087*X*, *R*^2^ = 0.9866 (Fig. 6c)). The FRET efficiency increased from 5.3 to 36.55% [29]. This result suggests that the quenching mechanism is sufficiently able to detect MNZ through the oxygen atom in MNZ that destroys the hydrogen bond between carbon quantum dots and carboxylated carbon nitride through competition, thereby destroying the FRET between N–C QDs and g-C_3_N_4_ nanosheets.

Previously reported works that detected MNZ in water media are compared in Table 1. The method fabricated in the present study has a lower detection limit. The response of the N–C QDs/g-C_3_N_4_ nanosheets to environmentally important common compounds such as l-histidine, lactose, oxalic acid, glucose, glycine, and starch was investigated under the same conditions to prove the selectivity of the method. No interference was observed when the concentrations of the substances were compared with the concentration of MNZ.

#### Possible Quenching Mechanism

**Fig. 7:**
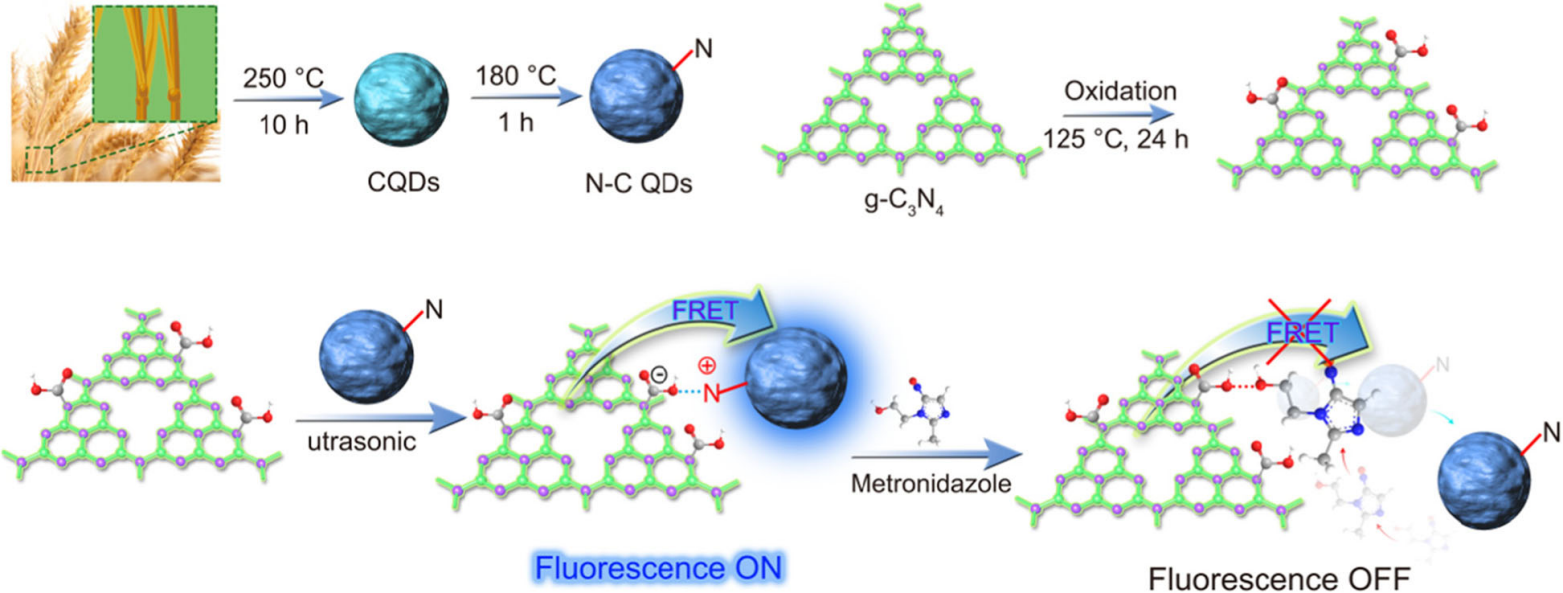
Schematic mechanism of Detection MNZ by g-C_3_N_4_/N–C QDs QDs composites

Figure 7 shows that we can observe that the fluorescence of g-C_3_N_4_/N–C QDs composites the complex can be quenched. A class of 0D/2D nanostructures based on N–C QDs and carboxylated g-C_3_N_4_ nanosheets was self-assembled (NˑˑˑH). The strong fluorescence of composites was effectively quenched by MNZ. MNZ exhibits an absorption spectrum in the range of 250 nm to 400 nm [30]. Intermolecular interactions between MNZ and nanosheets (electrostatic and hydrogen bonding between MNZ and nitrogen and terminal amino groups of nanosheets) destroyed the self-assembly between quantum dots and nanosheets at *λ*ex = 330 nm, the excitation of photoexcited electrons from nanosheets (as *π*− electron-rich donor) and from N–C quantum dots to MNZ (as electron-deficient nitroaromatic acceptor). The oxygen atom on the hydroxyl group in the MNZ structure has solitary pair electrons, and its electronegativity is greater than the nitrogen atom on the N–C QDs. The oxygen atom can destroy the N–H bond between g-C_3_N_4_/N–C QD composites and form O–H bond, thereby achieving the effect of fluorescence quenching of the system.

#### Recovery of MNZ in Water Sample

The water source near Xianyang Normal University was selected to investigate the feasibility of detecting trace MNZ in environmental water samples by fluorescence probe, and the recovery experiment was carried out. The concentration of melamine in water samples was determined by standard addition method under the optimized conditions. MNZ standard solution at 60 μL was added to the actual water sample for determination (*N* = 5). Results are shown in Table 2. The recovery of MNZ was 96.67–105.71%, which indicated that the method was suitable for determining MNZ in actual water samples, and the accuracy of the method was good [31]. These results show that the sensor has good accuracy and can be used for the detection of MNZ in biological samples.

**Table 2:** Recovery of metronidazole in different water samples (*N* = 5)

Water samples		Metronidazole solution (mol/L)	Recovery of metronidazole solution (mol/L)	Percent recovery
Distilled water		2 × 10^−5^	2 × 10^−5^	100%
Lake water		2 × 10^−5^	2.1 × 10^−5^	105.71%
Park canal		2 × 10^−5^	1.97 × 10^−5^	96.67%
Tap water		2 × 10^−5^	1.98 × 10^−5^	99.25%
Qinchi water		2 × 10^−5^	2 × 10^−5^	100.51%

A 0D/2D (0-dimensional/2-dimensional) nanostructures based on N–C QDs and carboxylated g-C_3_N_4_ nanosheets was designed as a FRET fluorescent sensor by self-assembly. At normal temperatures and pressures, N–C QDs/g-C_3_N_4_ nanostructures displayed good responses for the detection of metronidazole. The novel FRET sensor will has great potential in potential applications in clinical analysis and biologically related studies

## Conclusions

In this work, the 0D/2D nanostructure fluorescence measurement program realized the MNZ correlation according to its influence on the FRET process between the relative charged N–C QDs and the carboxylated g-C_3_N_4_ nanoparticles. N–C QDs/g-C_3_N_4_ nanocomposites can form FRET donors and receptor assemblies through electrostatic interaction, which effectively quenches the fluorescence emission of g-C_3_N_4_ nanocomposites. The FRET process is reduced mainly in the presence of the analyte because the oxygen atoms of MNZ destroy the electrostatic interaction assembly between N–C QDs/g-C_3_N_4_ (hydrogen bonds formed by nitrogen atoms from C QDs with hydrogen from carboxyl of g-C_3_N_4_ nanosheets), and the fluorescence emission decreases simultaneously. The method is easy to implement, easy to operate, and has high analytical scope and sensitivity. The fluorescence intensity of the assembled biosensor is proportional to the concentration of MNZ within − 2.6 × 10^−5^ mol/L under the optimum conditions.

## Supplementary information

**Additional file 1.** Highlights.

**Additional file 2.** Novelty.

## Data Availability

The data sets supporting the results of this article are included within the article and its additional files.

## Abbreviations

TEM: Transmission electron microscopy
PET: Photoinduced electron transfer
SV: Stern–Volmer equation
PNP: p-nitrophenol
UV–vis: Ultraviolet–visible spectroscopy
