# Supplementary material for: Highly Selective Detection of Metronidazole by Self-Assembly via 0D/2D N–C QDs/g-C3N4 Nanocomposites Through FRET Mechanism
Source: Nanoscale Res Lett. 2020 Apr 19;15:87. doi: 10.1186/s11671-020-3294-2 (PMC7167395; doi:10.1186/s11671-020-3294-2)
Supplement: Supplementary file 1 — Additional file 1. Highlights. [file 11671_2020_3294_MOESM1_ESM.docx]

Highlights

1 A 0-dimensional/2-dimensional nanostructures based on N-C QDs / g-C3N4 nanocomposites was designed as a FRET fluorescent sensor by self-assembly.

2 N-C QDs/g-C_3_N_4_ nanostructures displayed good responses for the detection of metronidazole. The fluorescence quenching equation is I = 1.0532+ 0.2087 C with the correlation coefficient R^2^ to be 0.9849. The MNZ concentration in a range from 0 to 2.6×10^-5^mol/L, and the detection limit was 0.66µM.

3 The FRET process is mainly because the oxygen atoms of metronidazole destroy the electrostatic interaction assembly between N -C QDs / g-C_3_N_4_ nanocomposites.
