# Supplementary material for: Highly Selective Detection of Metronidazole by Self-Assembly via 0D/2D N–C QDs/g-C3N4 Nanocomposites Through FRET Mechanism
Source: Nanoscale Res Lett. 2020 Apr 19;15:87. doi: 10.1186/s11671-020-3294-2 (PMC7167395; doi:10.1186/s11671-020-3294-2)
Supplement: Supplementary file 2 — Additional file 2. Novelty. [file 11671_2020_3294_MOESM2_ESM.docx]

Novelty

1 A 0-dimensional/2-dimensional nanostructures based on N-C QDs / g-C_3_N_4_ nanocomposites was designed as a FRET fluorescent sensor by self-assembly.

2 The fluorescence quenching equation is I = 1.0532+ 0.2087 C with the correlation coefficient R^2^ to be 0.9866. The detection limit of MNZ was 0.66 μM.

3 The FRET process is mainly because the oxygen atoms of metronidazole destroy the electrostatic interaction assembly between N -C QDs and g-C_3_N_4_ nanosheets
